# The susceptibility of the aortic root: porcine aortic rupture testing under cardiopulmonary bypass

**DOI:** 10.1186/s13019-021-01667-9

**Published:** 2021-10-03

**Authors:** Timothy Luke Surman, John Matthew Abrahams, Jim Manavis, John Finnie, Chris Christou, Georgia Kate Williams, Angela Walls, Peter Frantzis, Mark Adams, James Edwards, Michael George Worthington, John Beltrame

**Affiliations:** 1 D’Arcy Sutherland Cardiothoracic Surgical Unit, Royal Adelaide Hospital, Adelaide, SA Australia; 2 Cardiology Department, The Queen Elizabeth Hospital, Adelaide, SA Australia; 3 Department of Medical and Health Sciences, University of Adelaide Health Sciences, Adelaide, SA Australia; 4 Preclinical, Imaging, and Research Laboratories, South Australian Health and Medical Research Institute, Gilles Plains, Adelaide, SA Australia; 5 National Imaging Facility, Brisbane, Australia; 6 Dr Jones and Partners, South Australian Health and Medical Research Institute, Adelaide, SA Australia

**Keywords:** Aortic aneurysms, Cardiopulmonary bypass, Animal model, Histology, Wall sheer stress

## Abstract

**Background:**

In our earlier study on the functional limits of the aneurysmal aortic root we determined the pig root is susceptible to failure at high aortic pressures levels. We established a pig rupture model using cardiopulmonary bypass to determine the most susceptible region of the aortic root under the highest pressures achievable using continuous flow, and what changes occur in these regions on a macroscopic and histological level. This information may help guide clinical management of aortic root and ascending aorta pathology.

**Methods:**

Five pigs underwent 4D flow MRI imaging pre surgery to determine vasopressor induced wall sheer stress and flow parameters. All pigs were then placed on cardiopulmonary bypass (CPB) via median sternotomy, and maximal aortic root and ascending aorta flows were initiated until rupture or failure, to determine the most susceptible region of the aorta. The heart was explanted and analysed histologically to determine if histological changes mirror the macroscopic observations.

**Results:**

The magnetic resonance imaging (MRI) aortic flow and wall sheer stress (WSS) increased significantly in all regions of the aorta, and the median maximal pressures obtained during cardiopulmonary bypass was 497 mmHg and median maximal flows was 3.96 L/m. The area of failure in all experiments was the non-coronary cusp of the aortic valve. Collagen and elastin composition (%) was greatest in the proximal regions of the aorta. Collagen I and III showed greatest content in the inner aortic root and ascending aorta regions.

**Conclusions:**

This unique porcine model shows that the aortic root is most susceptible to failure at high continuous aortic pressures, supported histologically by different changes in collagen content and subtypes in the aortic root. With further analysis, this information could guide management of the aortic root in disease.

**Supplementary Information:**

The online version contains supplementary material available at 10.1186/s13019-021-01667-9.

## Background

In the realm of aortic root and ascending aorta aneurysm management, it remains unclear of their independent propensity to dissect or rupture under differing influencing factors. A number of animal models have been produced that have aimed to reproduce normal physiology (Table 1) however from our knowledge no animal model has replicated high aortic pressures beyond that of which is possible in human subjects to truly test the biomechanical limits of the aortic root and ascending aorta. Repetitive high continuous pressure and shear stress leads to a weakening of the aortic wall in susceptible patients resulting in an intimal tear [2], commonly in the lateral wall [3]. Biomechanical distinction between the aortic root and ascending aorta regions is scarce, yet clinical management of aortic root and ascending aorta pathology remains the same. Our objective is to use a porcine model to replicate the real time stresses placed on the aortic wall and aortic root apparatus under cardiopulmonary bypass and under the influence of vasopressor administration, to show the clinical and radiological effects of the aorta under stress, and determine the areas of greatest susceptibility to failure. We will determine the histological characteristics of acute stresses on the aortic wall between the ascending aorta and aortic root apparatus to determine if the macroscopic and microscopic changes align.

**Table 1:** Porcine models utilizing cardiopulmonary bypass

Author	Purpose	Methodology	Findings
Angelos et al. [4]	To determine organ blood flow changes in a swine model using CPB to achieve return of spontaneous circulation (ROSC)	Swine model of 10 pigs placed on CBP following VF cardiac arrest	Low flow cardiopulmonary bypass model produces reproducible high resuscitation rates and ROSC
Bufalari et al. [5]	To determine the most effective practice of left pneumonectomy	Swine model of 11 pigs undergoing left pneumonectomy	The most straightforward procedure required careful dissection of the pulmonary ligament, pulmonary veins, pulmonary artery, and finally bronchus
Eckhouse et al. [6]	To establish a reproducible model of aortic dilatation reproducing what happens in Thoracic abdominal aneurysm’s (TAA) development	Descending TAA’s were induced in 7 pigs using collagenase and crystalline and tissue analysed	Tissue demonstrates aortic dilatation, aortic medial degeneration, and alterations in MMP/TIMP abundance consistent with TAA formation
Kofidis et al. [7]	To determine the feasibility of transapical cardioscopic surgery in a pig model	Transapical access to the ventricle was obtained in 5 pigs with right mini thoracotomy for central cannulation and CPB	Transapical approach allowed for good exposure and adequate surgical field for mitral valve, and aortic valve access, and atrial ablation and intra-aortic procedures
Lundemeon et al. [8]	To determine the effects of pulsed and non-pulsed CPB on microvascular fluid exchange	A total of 16 pigs were randomized to pulsatile (n = 8) or non-pulsatile (n = 8) CPB	No significant differences in the fluid extravasation rates were present between pulsed and non-pulsed cardiopulmonary bypass perfusion
Mariscal et al. [9]	To describe a surgical technique for swine lung transplantation and postoperative management 3 days postoperatively	Involved development of a protocol based on donor surgery, recipient surgery and postoperative care and sacrifice	This survival model can be used by lung researchers to assess development of primary graft dysfunction (PGD) and to test therapeutic strategies targeting PGD
Mickelson et al. [10]	To develop an alternative to canine models in testing for cardiopulmonary bypass research	15 pigs were divided into three groups to determine the optimum conditions during CPB to avoid complications of fluid shifts, metabolic acidosis, and hemoglobinuria	Determined that optimum blood flow rate for cardiopulmonary bypass in swine is in the range of 175–200 ml/kg min. Hyperosmolar priming solution is beneficial for CPB in swine to reduce fluid shifts, metabolic acidosis, and hemoglobinuria
Nicols et al. [11]	To determine the effect of changing FiO2-concentration on SvO2 in a swine model on CPB	8 mixed-gender swine were placed on CPB with an experimental and control group measuring percentage change in blood flow and oxygen delivery	Results suggest that decreased blood flow adjusting for increased SvO2 associated with high PaO2 did not result in significant reduction in adequacy of perfusion markers for organs studied
Oizumi et al. [1]	Development of a swine model for anatomical thoracoscopic lung segmentectomy training	33 pigs were used over a period of 5 years to train operators on segmentectomy via a hybrid (8) or thoracoscopic (23) approach. 3 pigs were converted to thoracotomy due to hemorrhage	Live swine model was considered a good choice for training surgeons on how to perform a minimally invasive lung segmentectomy in humans
Thalmann et al. [13]	Evaluation of several hybrid approaches for pulmonary valve replacement in a swine model	13 pigs were used using 4 different thoracotomy methods for valve implantation, and 5 cases used median sternotomy	Achieved implantation of 12/13 stented valves of which 41% were in the optimal position and 16% had paravalvular leakage. Lower partial sternotomy provided the best deemed approach

## Materials and methods

All investigators complied with the 2011 "Guide for the Care and Use of Laboratory Animals", and approval by the South Australian Health and Medical Research Institute Animal Ethics Committee (SAHMRI AEC).

### Animal preparation

Following our pilot study indicating differences between the rupture potential of the aortic root and ascending aorta in porcine aortas [1], 5 female adult pigs were obtained for animal testing. All pigs weighed between 50 and 60 kg and were in good health. All animals had external jugular vein and carotid arterial monitoring placed 2 days prior to the testing. Pigs underwent induction using 3–5 ml intramuscular ketamine, maintenance using 2–3% isoflurane, with ongoing ventilation and flow rate of 3–4 L/min. Ongoing monitoring of mean arterial pressure (MAP), systolic and diastolic blood pressure, heart rate and end title CO2 (etCO2) occurred with all experiments with observations recorded every 15–20 min.

### Preoperative MRI imaging

All pigs underwent baseline MRI imaging at normal blood pressure and heart rate hemodynamics. All pigs then received a bolus noradrenaline dose of 5–6 ml at 4 mg/4 mL until systolic blood pressure exceeded 200 mmHg. Each pig then underwent MRI at systolic pressures > 200 mmHg to measure WSS and flow parameters.

**Fig. 1:**
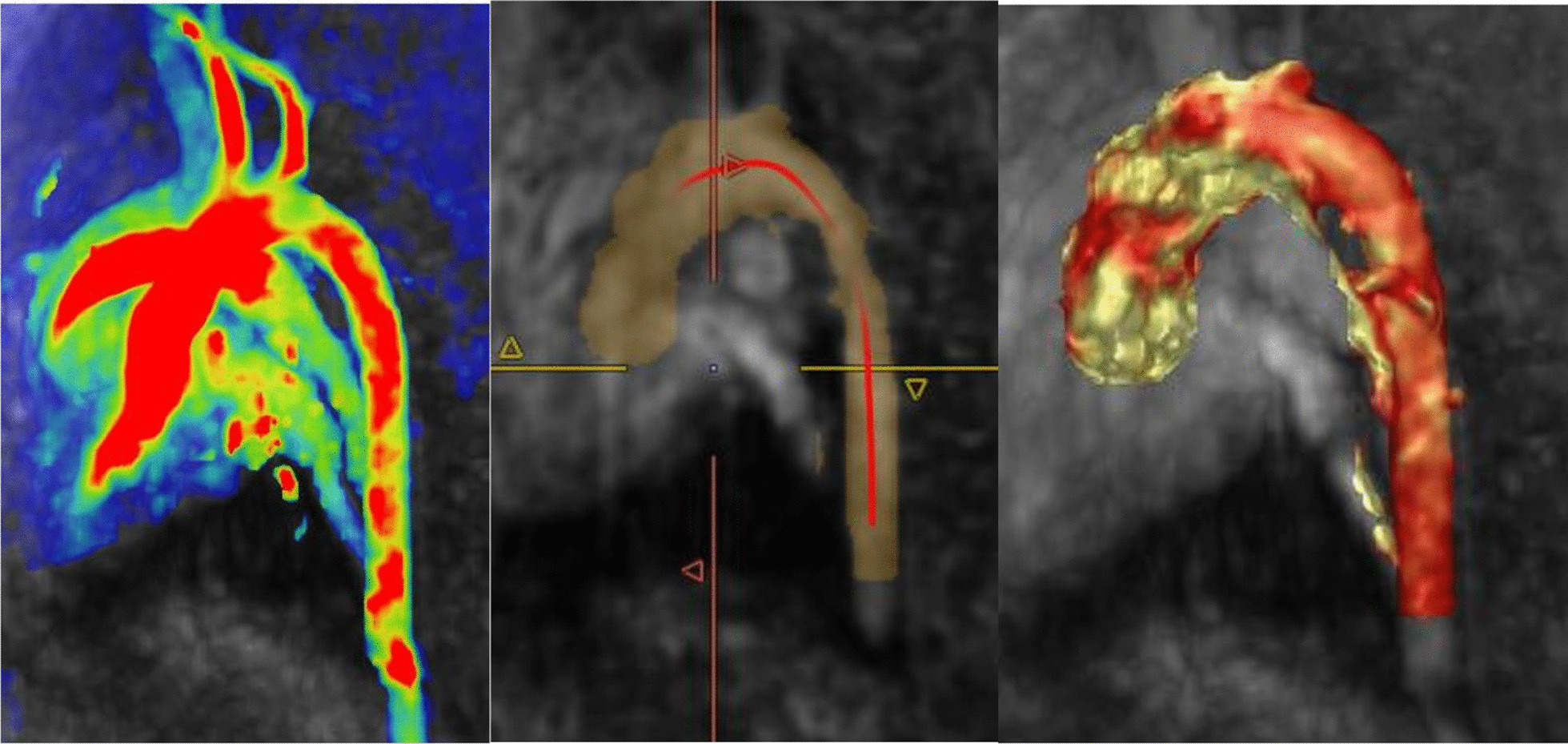
Porcine subject 4D flow MRI preprocessing (left), segmentation (middle), and the aorta ready for analysis (right) as performed using Circle CVI42 version 5.10.1

All MRI scans were performed using a 3-Tesla Siemens Magnetom Skyra (Siemens Healthcare, Erlangen, Germany) (Fig. 1). The subject was positioned in dorsal recumbency within a custom-made MRI compatible positioning device. The subject’s condition during MRI was monitored using invasive blood pressure monitoring and an MRI-safe pulse oximeter. Siemens Works In Progress (WIP) sequence, 4D Phase Contrast Flow (WIP 785A) was employed to quantify time-resolved flow within the aorta through cartesian sampling in three dimensions. The MRI images were analysed using Circle Cardiovascular Imaging (CVI42) version 5.10.1 Inc, Calgary, Alberta, Canada (Table 2). Each pig study underwent data cropping to identify the area of interest which included the aortic root, ascending aorta, arch and descending aorta. The selected area then underwent preprocessing, whereby a tissue mask is defined. Offset correction and phase anti-aliasing was applied if unwanted flow or noise was identified. The vessel was then segmented, by tracing a centerline from the aortic valve to the descending aorta of which measurement will be determined, and vessel diameter mask adjusted until appropriate for the size of the aorta. Analysis then began with flow measurements. A flow plane is positioned along the center-line until at the appropriate level on the aorta. Each flow plane was positioned at the aortic root, proximal ascending aorta, middle ascending aorta, and distal ascending aorta in which measurements would be taken. Adjustments were made using double oblique views until cross-sectional images were accurately displayed and flow planes aligned. Each measurement was added and flow calculation determined. Net flow (ml/cycle), Peak velocity (cm/s), and regurgitant flow (k) values were calculated automatically. Using the same anatomical plane, wall sheer stress was automatically calculated. Axial maximum WSS (Pa) and Axial average WSS (Pa) was determined.

**Table 2:** MRI phase contrast flow parameters

Siemens Skyra 3T 4D phase contrast flow parameters	
Field of view (FOV)	390 mm × 266 mm
Matrix	176 × 141
Voxel size	2.2 mm × 2.2 mm × 2.2 mm (isotropic)
Repetition time (TR)	40.32 ms
Echo time (TE)	2.29 ms
Velocity encoding (VENC)	180
flip angle	8°
Gating	Retrospective cardiac
Coils	Spine matrix and 18-channel body array

### Animal operation

**Fig. 2:**
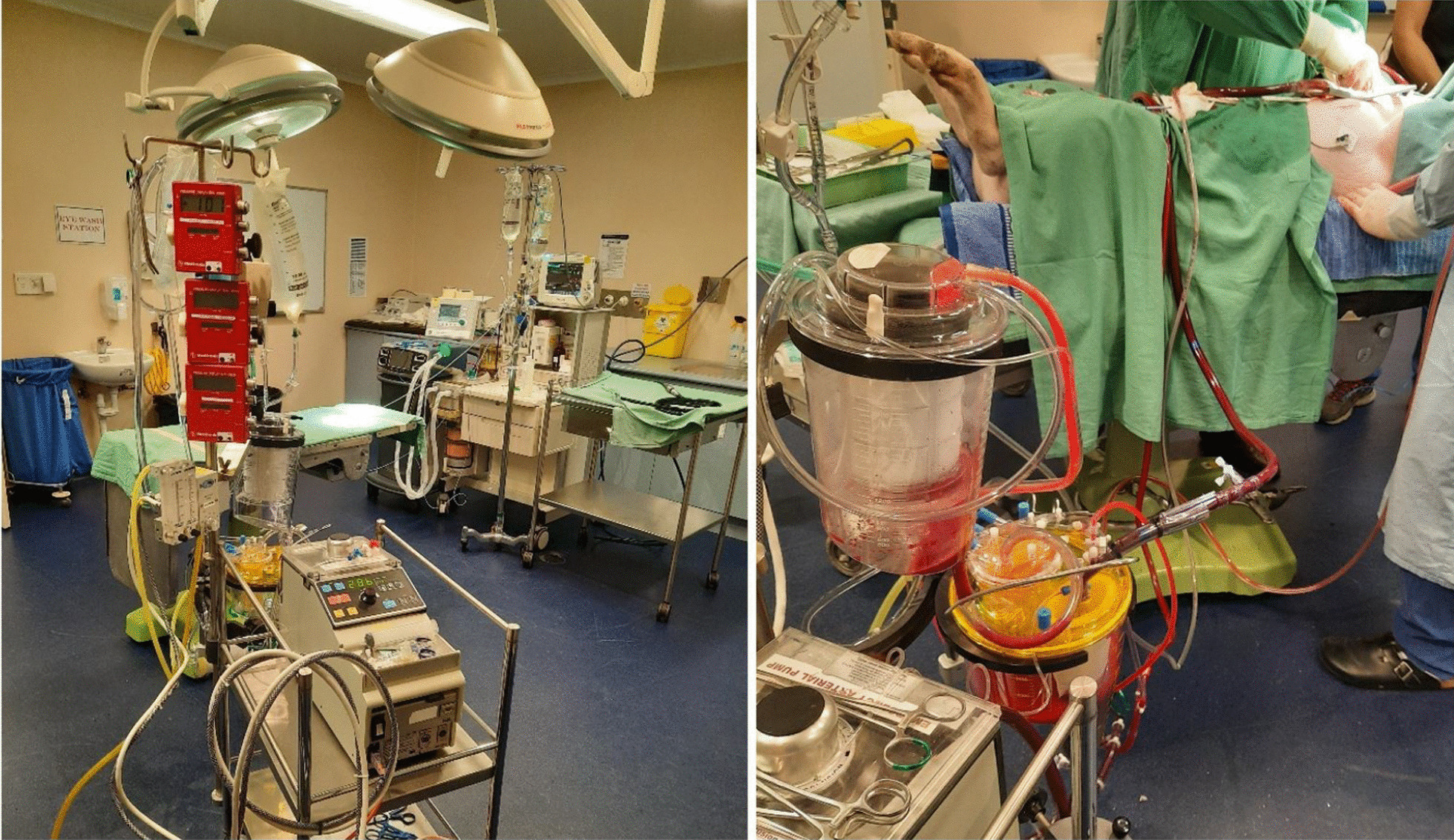
Cardiopulmonary bypass circuit setup for porcine testing (left), and the active CPB circuit during the porcine experiments (right)

**Fig. 3:**
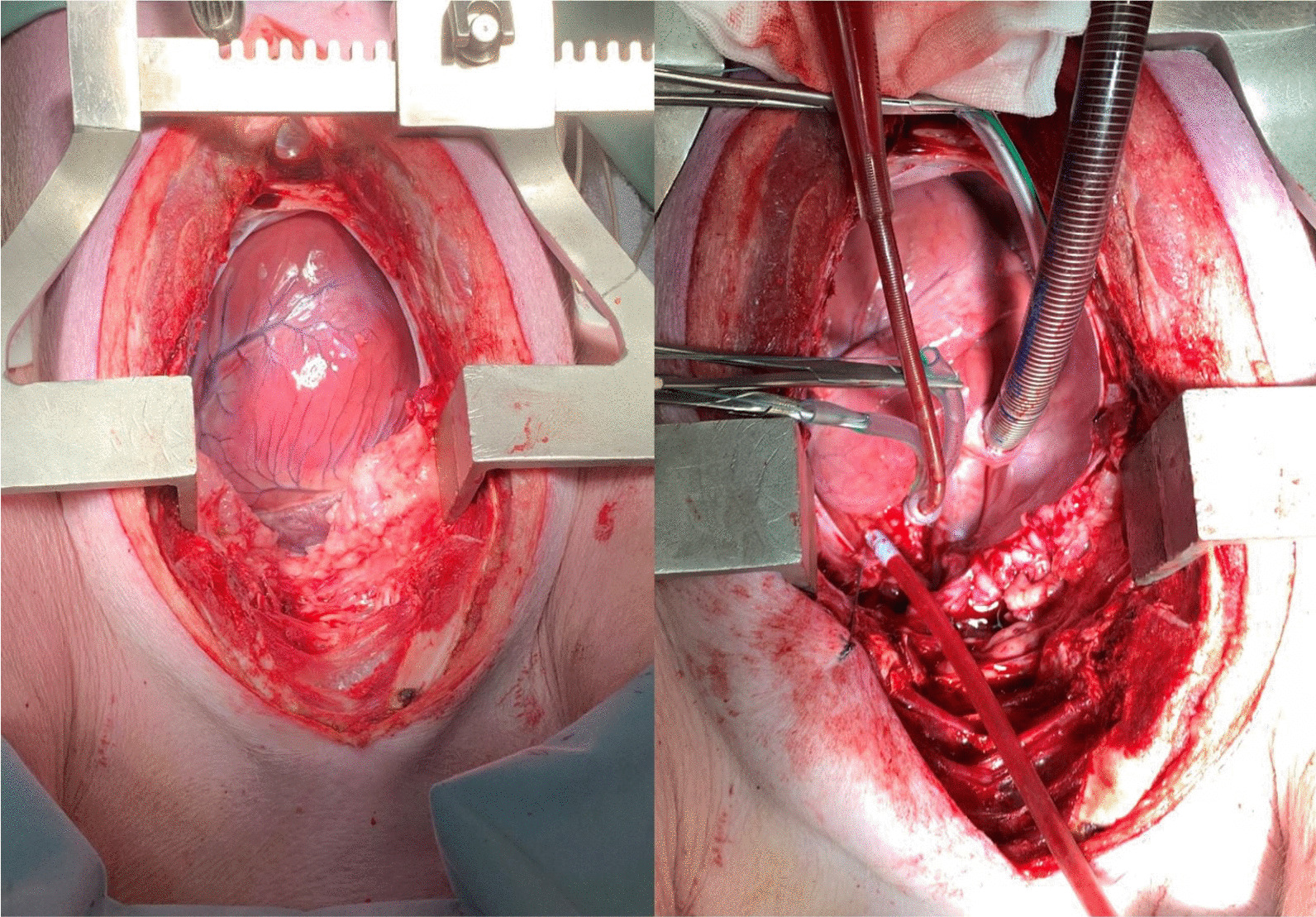
Median sternotomy and porcine heart exposed (left), and establishment of central cardiopulmonary bypass with porcine subject (right)

Following MRI imaging and normalization of pig hemodynamics including heart rate and blood pressure, a cardiopulmonary bypass circuit was created to replicate an adult circuit with a cardiac perfusionist managing its function. Two veterinary assistants monitored and managed the pig throughout the process. Two surgeons were the primary operators for each pig. For all experiments, cardiopulmonary bypass (LivaNova Circuit) was utilized, prepared with a roller pump and inspire oxygenator. Three-eighths tubing was used to replace the pump header, attached to the autolog reservoir and inspire cardiotomy reservoir and clamped off. Two suckers were utilized for the operative field and primed with 25,000 IU of heparin in 1000 ml of saline. The CPB circuit was primed with 1.6L of saline and 5000 IU of heparin. Operation time for each pig was between 60–120 min. Direct anterior access via a median sternotomy proved to give best access to the aorta and right atrium for cannulation (Figs. 2 and 3). Following heparinization of 15,000 IU, the right atrium was cannulated using a 32f Medtronic venous canula, and the ascending aorta cannulated with a 16f Edwards Lifesciences cannula. Bypass was initiated with good flows, with incremental increases in pressures over the next 10 min. A cross clamp was applied at the distal arch. Cardiopulmonary bypass flows were then increased to maximal flows (L/min) and line pressures, and kept at these measures for 60 s with ongoing monitoring until aortic or cardiac failure. Cardiopulmonary bypass was ceased and euthanasia was performed with 20 ml of intravenous phenobarbitone overdose.

### Macroscopic and histological analysis

The aorta was carefully dissected from the left ventricle to the start of the aortic arch in all pigs. Careful attention was made to handling the aorta to ensure no tissue damage was inflicted in this process. from the pig and examined by the two operating surgeons. The aortic root, and ascending aorta were then cut into aortic root, proximal, mid, and distal regions.

Tissue was immediately placed in formalin for fixation following preparation, embedded, and cut using a Leica rotary microtome (Leica Biosystems, Mt Waverley Australia) into 5micro-metre edge-to-edge sections. The basic histological stains and special stains used included Hematoxylin and Eosin (H&E), Van Gieson (EVG), and Massons Trichrome (Massons), Alcian blue, and Von Kossa (VK) stains. Specific immunochemistry antibodies staining for Collagen type I, III and IV were obtained from Abcam Australia Pty Ltd (Melbourne, Victoria, Australia). Anti-Collagen I antibody, Anti-Collagen III antibody, and Anti-Collagen IV antibody were sourced.

Observational analysis proceeded with the primary investigator and a clinical histopathologist. Histological analysis occurred with the use of a double headed microscope at the University of Adelaide Histological department, Adelaide, South Australia.

Histological slides were scanned using Nanozoomer digital slide scanner (Hamamatsu Photonics), Zen Blue 3.0 (Zeiss) and NDP view 2.0 (Hamamatsu Photonics) depending on the slide size. Scanned histological slides were then analysed using Fiji by Image J (National Institutes of Health, USA).

Quantification of elastin and collagen fibers then proceeded using the colour deconvolution plugin (IHC toolbox) in Image J v.1.53 (The University of Nottingham, UK). The image was imported into Image J from the NDP or Zen programs, the image cropped to select a region of interest (ROI), and then colour deconvoluted. This ROI then underwent analysis and measurement in Image J to produce a percentage quantification of collagen fibers or elastin fibers within that tissue specimen.

## Results

### Clinical results

The clinical results from the 5 porcine studies are summarized in Table 3. Median maximal aortic pressures obtained amongst the tested samples was 497 mmHg. The median maximal CPB flows (L/min) was 3.96L. The most common macroscopic findings were aortic cusp hemorrhage and non-coronary cusp tearing which occurred in 4/5 samples (80% of tested cases).

**Table 3:** Clinical results and macroscopic findings following maximal aortic pressures on CPB

Swine number	Surgical approach	Cannulation	CPB flows (L/min)	Maximal pressure	Macroscopic findings
1	Right thoracotomy	Arterial—ascending aorta Venous—Right atrium	2L	280 mmHg	Valvular failure with no evidence of cusp tearing Cusp hemorrhage present Superior Vena Cava (SVC) tearing resulting in exsanguination of subject
2	Median sternotomy	Arterial—ascending aorta Venous—right atrium	2.2L	286 mmHg	Non-coronary cusp tearing and valvular rupture Cusp hemorrhage Subject euthanized
3	Median sternotomy	Arterial—ascending aorta Venous—right atrium	4.3L	500 mmHg	Non-coronary cusp tearing and valvular rupture Cusp hemorrhage Subject euthanized
4	Median sternotomy	Arterial—ascending aorta Venous—right atrium	5.4L	505 mmHg	Non-coronary cusp tearing and valvular rupture Subject euthanized
5	Median sternotomy	Arterial—ascending aorta Venous—right atrium	3.96L	497 mmHg	Non-coronary cusp tearing and valvular rupture Cusp hemorrhage Subject euthanized
Median			3.96L	497 mmHg	

### Radiological results

The median max flow (cm/s) in all samples was 79.05 at baseline, and 95.53 following vasopressor. The median wall sheer stress (WSS) (Pa) in all samples was 0.31 at baseline, and 0.48 following vasopressor (Additional file 1: Table 1).

**Fig. 4:**
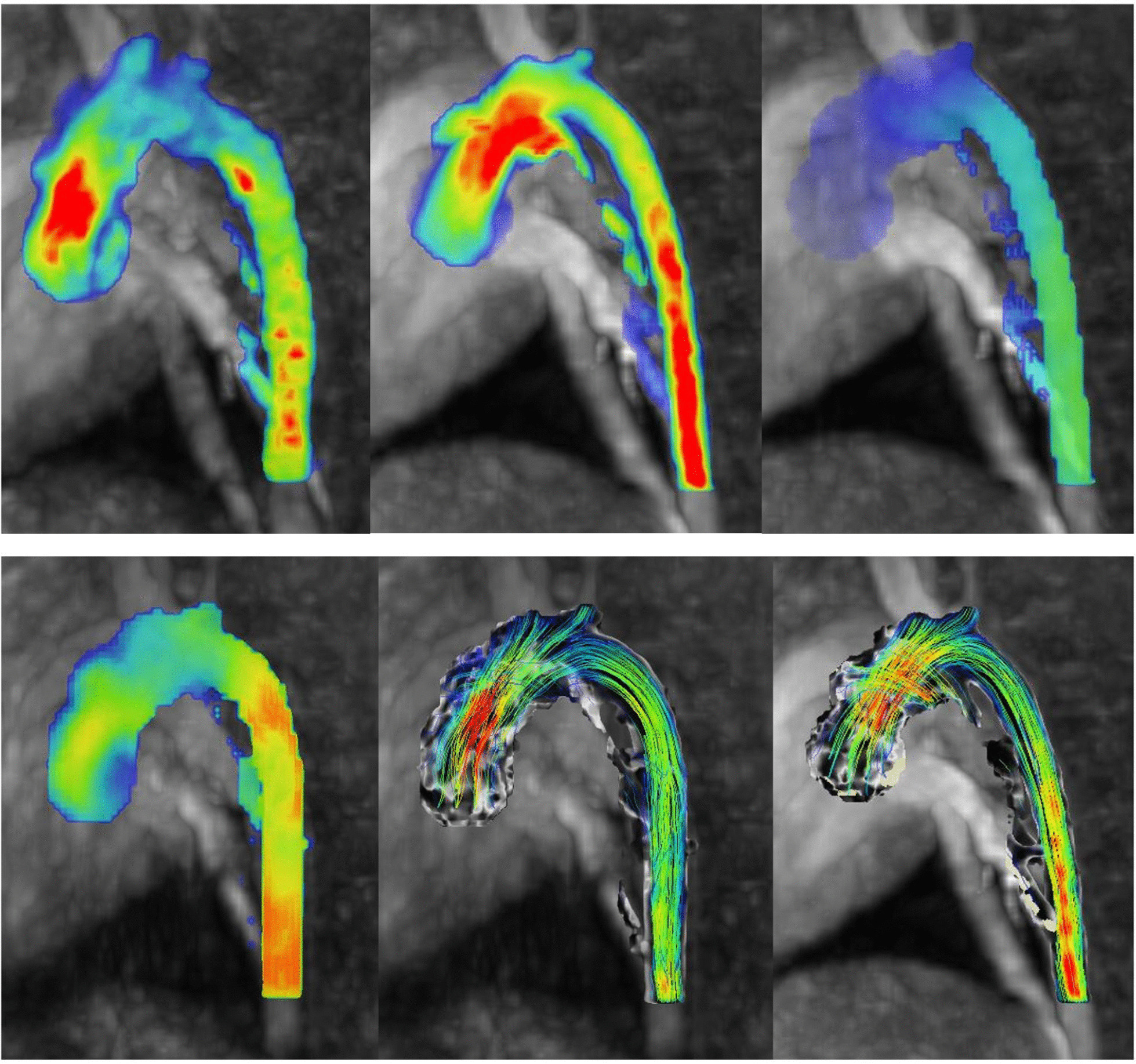
4D flow MRI imaging results in the porcine subjects. Top left—Porcine flow measurements pre-administration of noradrenaline and Top centre—porcine flow measurements post-administration of noradrenaline. The red shading indicates areas of higher flow measurements (cm/s). Porcine wall sheer stress measurements. Top right—porcine WSS measurements pre-administration of noradrenaline and Bottom left—porcine WSS measurements post-administration of noradrenaline. The areas of yellow-orange-red identify regions of higher WSS (Pa) in ascending order in the porcine subject. Bottom centre—Porcine pathline results pre-administration of noradrenaline and Bottom right—porcine pathline results post-administration of noradrenaline. The pathlines show the direction of blood flow during these stages

The median max flow (cm/s) at baseline in the aortic root was 53.90, and 64.12 following vasopressor. Median flow in the proximal ascending aorta at baseline was 74.73 and 88.58 following vasopressor. Median flow in the middle ascending aorta at baseline was 84.70 and 101.33 following vasopressor. Median flow in the distal ascending aorta at baseline was 86.37, and 101.95 following vasopressor (Fig. 4) (Additional file 1: Tables 2 and 3).

The median WSS (Pa) at baseline in the aortic root was 0.23, and 0.35 following vasopressor. Median WSS in the proximal ascending aorta at baseline was 0.32 and 0.49 following vasopressor. Median WSS in the mid ascending aorta was 0.37 at baseline, and 0.59 following vasopressor. Median WSS in the distal ascending aorta was 0.31 at baseline, and 0.45 following vasopressor (Additional file 1: Tables 4 and 5).

Although not a direct measure within our study cohort, observational analysis of pathlines pre- and post-administration of vasopressor showed increased vortices flow within the ascending aorta following the administration of vasopressor (Fig. 4).

### Histological results

**Fig. 5:**
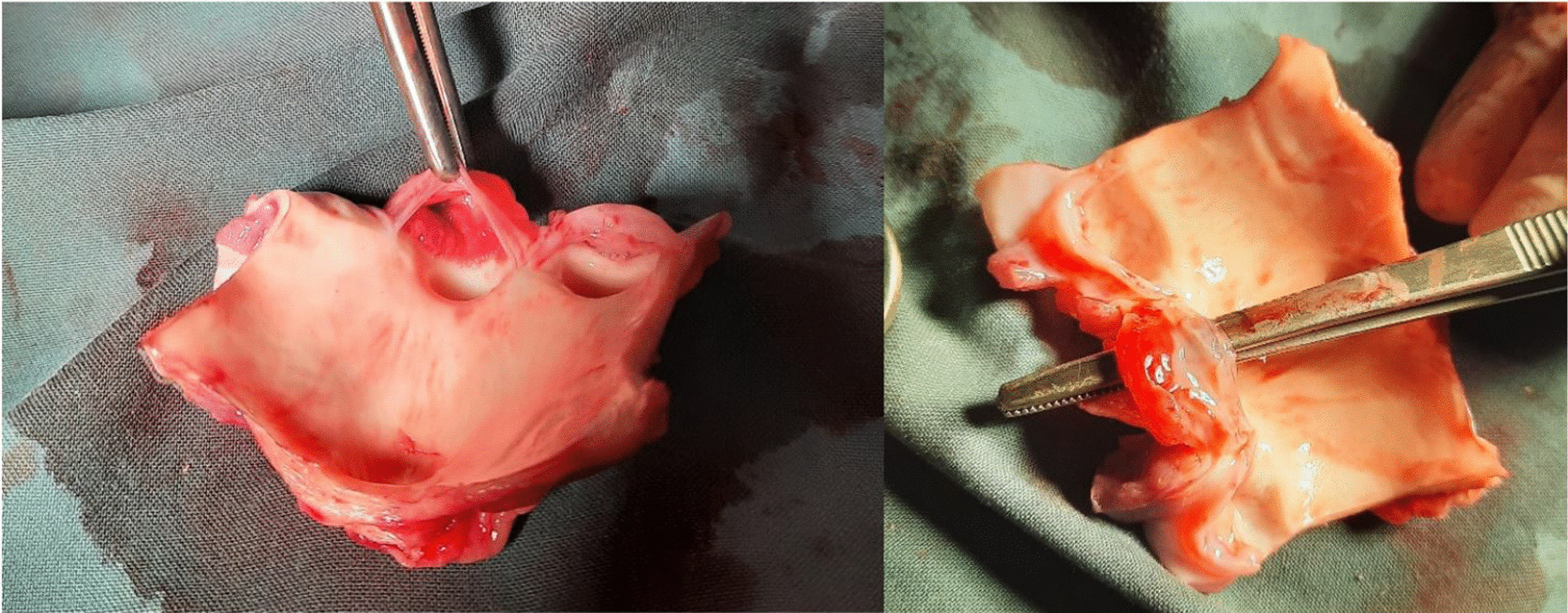
Photographs of the excised and opened aortic root identifying the tears beneath the non-coronary cusp within each porcine subject tested

Large tears beneath the non-coronary cusp were noted in all samples (Fig. 5).

**Fig. 6:**
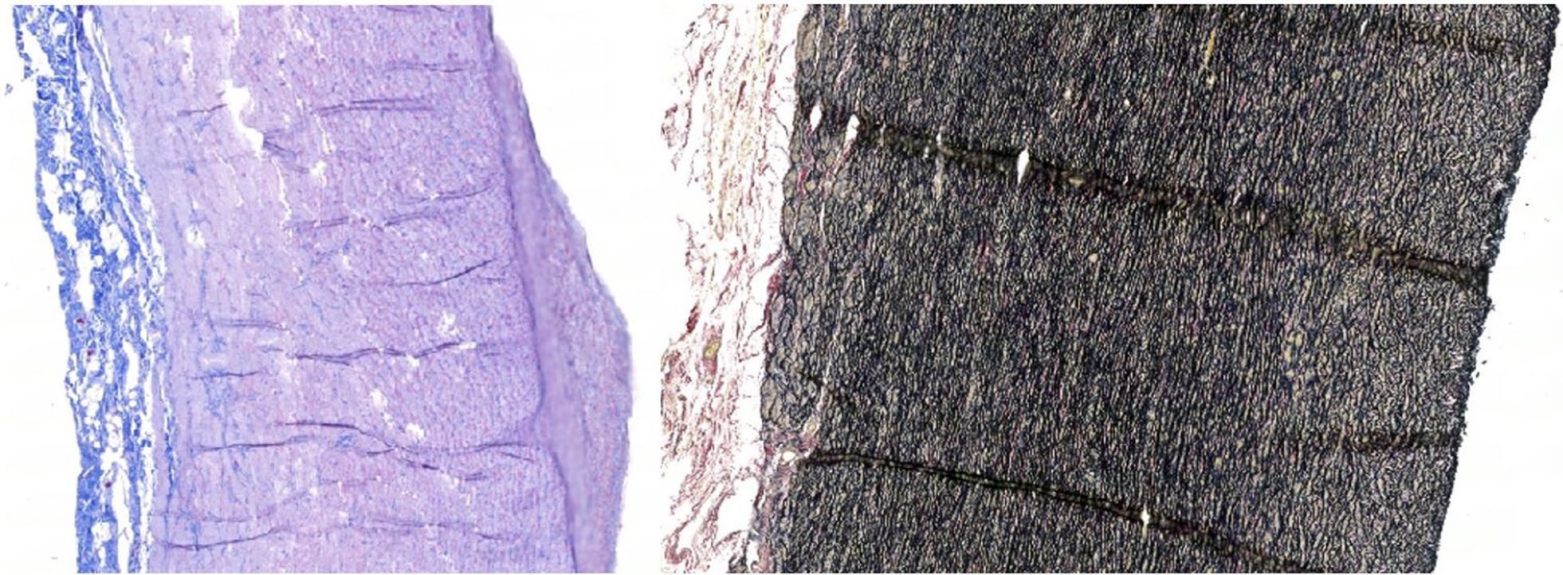
10 × Massons trichrome staining of the porcine aortic root with darker blue areas indicating collagen deposition (left image). 10 × Van Gieson (EVG) staining of the porcine aortic root with black areas indicating elastin deposition (right image)

The average collagen composition (%) was highest in the proximal inner region (8.48) and proximal outer region (9.08); with other regions having approximately half that of the proximal regions. The average elastin composition (%) was highest in the proximal inner region (23.10). Elastin content was also high in distal and middle inner regions and the aortic root itself compared to other regions (Additional file 1: Tables 6 and 7) (Fig. 6).

**Fig. 7:**
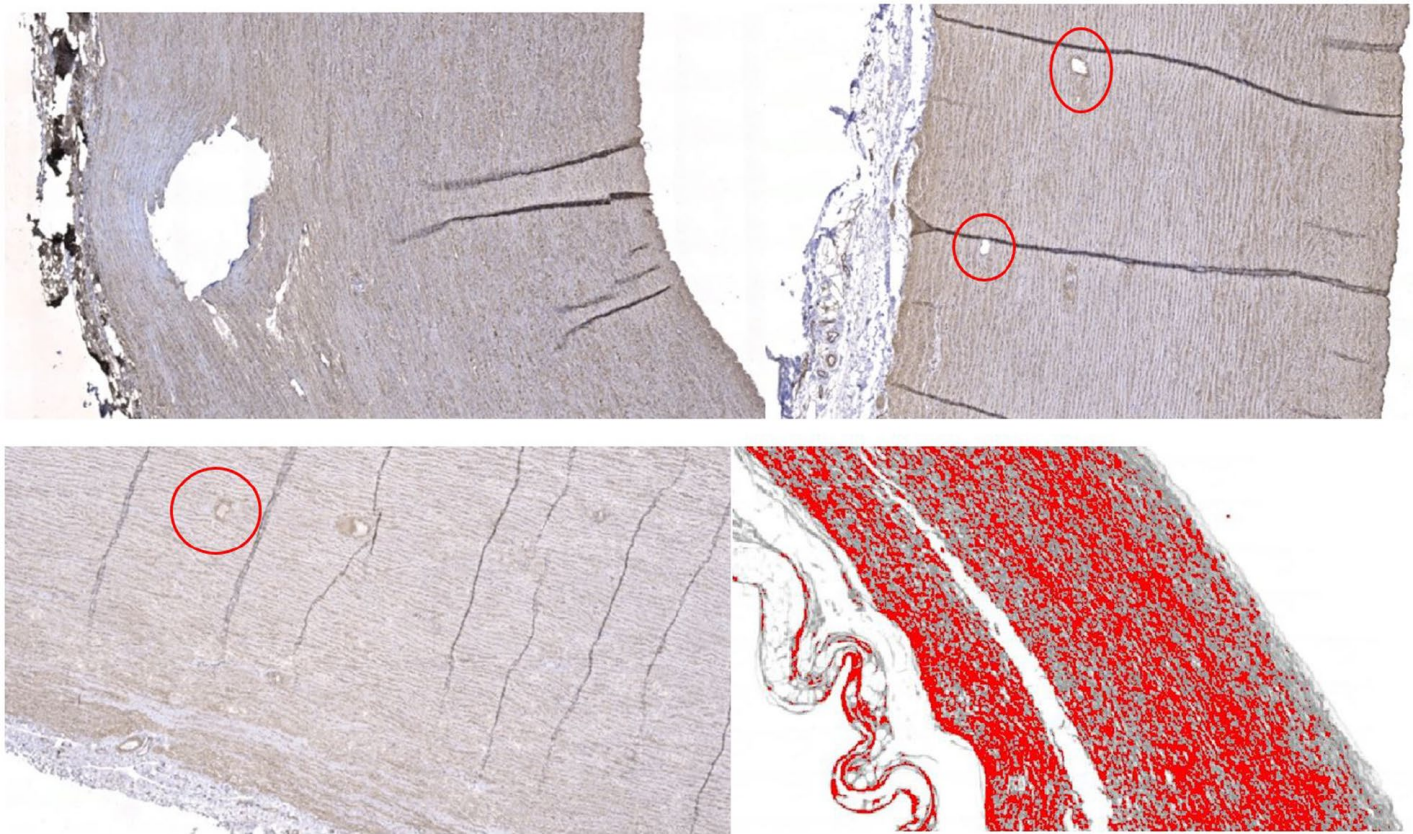
Immunohistochemistry results showing collagen types within the porcine aortic root and ascending aorta. Top left—Collagen I staining within the porcine proximal aorta as indicated by the brown staining. Top right—Collagen IV antibodies within the porcine ascending aorta noting the positive internal structure staining of blood vessels as highlighted. Bottom left—Collagen IV antibodies within the porcine ascending aorta with positive staining of internal blood vessels as highlighted. Bottom right—Colour deconvolution of immunohistochemistry results showing quantification of Collagen I in the proximal porcine aorta as highlighted by the dense red areas

General observations were loss of tissue architecture in the aortic root and microhemorrhages in the non-coronary cusp region in all subjects. Immunohistochemistry observations of Collagen I stained specimens showed stronger staining under the intimal layer in all subjects. Collagen III analysis showed diffuse and weak staining in all subjects. Collagen IV analysis showed gross staining with positive blood vessel internal markers within the aortic root in all specimens (Fig. 7).

The average collagen I composition (%) was highest in the distal inner region (28.92), followed by the middle inner (26.15), and proximal outer (25.75) regions. Collagen I was also high in the aortic root (24.53). The average collagen III composition (%) was highest in the middle inner (25.29) and aortic root regions (23.68). The median collagen IV composition (%) was highest in the middle outer (24.51) and proximal anterior (22.35) aorta (Additional file 1: Tables 8–10).

## Discussion

The aim of this study was to determine what area of the aortic root was most susceptible to failure at high aortic pressures, and how these pressures manifest radiologically and histologically in acute rupture. Using a physiological model, results could identify patterns in radiological, pathological, and histological changes that affect the aorta under stress. Although no studies apart from Surman et al. [1] have reported on the maximal pressures obtained in the porcine aortic root and ascending aorta, many studies have showed that a porcine model is effective in the application of cardiopulmonary bypass and replicating models applicable to human subjects in cardiothoracic surgery [4–13].

Intimal tears are reported to occur mostly in the right lateral wall of the ascending aorta in humans [3], however studies reporting on the most common sites are not well described. Tears affecting the proximal ascending aorta and distal arch have the most catastrophic consequences as they compromise the heart, and brain, respectively. Although our porcine subjects were not aneurysmal, not all dissections and aortic rupture occurs in aneurysmal patients, and therefore the results hold pathological value in interpretation. When it came to location of the tears, all porcine subjects had splitting beneath the non-coronary cusp and aortic valve failure, identifying it as an area of weakness under high continuous aortic stresses. Surman and colleagues [1] found that the aortic root apparatus in porcine subjects failed at lower pressures compared to the ascending aorta, identifying a clear difference between these two tissues. Clinical findings in this study, supported those findings with failure of the aortic valve apparatus and preservation of the ascending aorta in all regions.

We examined the impact of high intraluminal pressures on the aorta using 4D flow MRI. Median flow measured in cm/s increased significantly, and WSS almost doubled on average across all subjects in all regions of the aorta, identifying that high stresses manifest throughout the aorta from root to distal ascending in only an acute period of time. When we examine the regional changes, the proximal, middle, and distal ascending aorta had significant increase in flow following vasopressor administration indicating that this distribution of increased flow propagates from the root to the arch. Even more profound, was that WSS (Pa) almost doubled in all regions of the aorta following vasopressor administration. The increase in aortic stress was greatest in the mid ascending aorta but high in all regions from the root to the arch. The increase in WSS also correlates to the WSS showing highest increases in the mid and distal ascending aorta groups.

When we review the acute immunohistochemistry and histological changes that result from these acute stresses, we have to determine what is normal before comparing to what is abnormal. The two main types of collagen found in the aorta are types I and III and account for 80–90% of the total collagen, and remaining collagens in lesser amounts [14]. Collagen staining of types I and III was more intense in cases of TAA dissection than in controls and were characterized by thick longitudinal sheets or bundles in the media which were larger than type IV [14, 15], while othersshow collagen proportion in the wall of the dissected and aneurysmal TAA was less than control [16, 17]. Histological and immunohistochemistry analysis in a swine model is not reported in the literature. Interestingly we found that Collagen type I had quite intense staining throughout the intimal layers in all specimens, whereas type III was less abundant. Type IV collagen is less abundant but in control TAA and normal histological samples of TAA dissection, type IV collagen were seen between the subintimal basement membrane and the media, and in the basement membrane of the adventitia [14].

In our study, collagen IV was prominent in the proximal ascending and aortic root compared to other regions. Eckhouse and colleagues [6] in thoracic abdominal aneurysms in pigs, reported aortic structural changes includingelastic lamellar degradation and decreased collagen content. and colleagues [18] examined differences in aortic sinus tissues between human and pigs. The porcine tissues contain a higher proportion of elastin than the human tissues, which contain a higher proportion of collagen. The elastin fibers in the porcine tissues also appeared to be more undulated than the elastin fibers in the human samples, which were thinner and straighter. This study is limited by the use of a single special stain and lack of quantification of their findings. Collagen I was clearly higher within inner regions across proximal, middle, and distal aortic areas, and similarly collagen III was highest within inner regions including the aortic root. Collagen IV as the least commonly reported type in the thoracic aorta was more equally distributed across regions but showed some higher content in the more middle and proximal regions of the ascending aorta.

Determining protein quantification in porcine tissue is scarce in the literature. A study in 1985 from Davidson and colleagues [19] aimed to determine this in newborn pigs. Relative collagen and elastin syntheses, as a per cent of total protein synthesis, were determined in four separate experiments. Elastin synthesis decreased from about 16.4% in the thoracic aorta to 1.6% of total protein synthesis in the abdominal aorta. Collagen synthesis showed the opposite trend, increasing to 12% of total protein synthesis, although collagen synthesis was still a significant fraction (5–8%) of total protein synthesis in the upper thoracic tissue [19]. Collagen composition was reported as higher in the proximal inner and outer regions of our samples on average across all specimens. Elastin composition was also recorded highest in the inner regions across proximal, middle, and distal aortic regions.

This detailed live animal modelling under conditions of ongoing continuous flow have revealed some important information regarding acute aortic pathology. We have determined that area of greatest risk of failure during high pressure and flow conditions is the non-coronary cusp of the aortic valve within the aortic root apparatus as confirmed by macroscopic and microscopic findings. We have found that the regions of the thoracic ascending aorta under greatest WSS after increased vasopressor insult is the proximal and middle ascending aorta regions.

Histopathology analysis has revealed that the proximal and inner regions of the thoracic ascending aorta have collagen and elastin content that differs from the remaining aortic structure which may predispose or protect it from more chronic insults. When it came to specific collagen content as measured by immunohistochemistry, proximal and inner regions similarly had high collagen I, III, and IV levels but specifically the aortic root had some of the highest collagen I and III levels within the tested samples. We determined that Collagen IV was actually quite a dominant figure in the ascending aorta alone, but was found in minimal amounts in the aortic root.

When we compare the histological and immunohistochemistry analysis of non-aneurysmal samples in pigs and humans there are similarities between quantification values as reported in an upcoming article for publication by Surman and colleagues. When we compare human aneurysmal collagen and elastin quantities, the values are similar between human aneurysmal elastin content and porcine elastin in this study, but the collagen content differs considerably. When we review the human aneurysmal immunohistochemistry versus porcine values in this paper, we see significant differences. The quantity of Collagen I, III and IV are all significantly reduced in human aneurysms compared to non-aneurysmal acute ruptured porcine samples. Reassuringly there is good reproducibility of quantification between porcine and human non-aneurysmal samples as shown in earlier studies [4–13].

Limitations in this study includes histological analysis, whereby immunohistochemistry techniques are limited by the ability of the tissue to take up by the antibodies in question which result in more difficult specimens to analyse and quantify. In addition, pathological analysis and quantification are limited by the investigator and varies considerably with each analysis. This is supported by the results of the same sample shown in the Additional file 1: Tables which shows variation in final percentages following analysis by each investigator of the same sample. Limitations also include the surgical approach. Access and initiation of cardiopulmonary bypass is very challenging and this was shown by difficulties in initial attempts at surgical access. The authors agree that a median sternotomy approach to the ascending aorta is best. Limitations in MRI include long acquisition times, parallel imaging techniques used to compensate (i.e., decreased special and temporal resolution), and the sequence is a WIP so is still investigational.

To our knowledge, this is the first live porcine study measuring the limits and resulting pathology of the aortic root and ascending aorta under high pressures during cardiopulmonary bypass supported by earlier pilot ex-vivo studies [1]. Similarly, no study has quantified the microscopic details of the aortic root and thoracic ascending aorta following such acute insult.

## Conclusion

We have identified that the most vulnerable structure in the aortic root apparatus is the non-coronary cusp of the aortic valve, and the aortic root itself reveals histopathological characteristics such as collagen content and collagen types that differ from the ascending aorta itself, supporting by upcoming histological analysis of human subjects by Surman et al.

These findings further support the idea that the aortic root apparatus, extending up to the inner proximal ascending aorta need to be considered as an independent structure with unique structural susceptibilities and limitations, and protein composition and that should be considered a more vulnerable and delicate structure in surgical management. Further live animal testing in aneurysmal aortas would provide valuable details to these results.

## Supplementary Information

**Additional file 1.S1**: Summary of radiological results following noradrenaline administration and 4D flow MRI imaging. **S2**: Regional analysis of Flow (cm/s) in 4D flow analysis pre-vasopressor administration. **S3**: Regional analysis of Flow (cm/s) in 4D flow analysis post vasopressor administration. **S4**: Regional analysis of WSS (Pa) in 4D flow analysis pre-vasopressor administration. **S5**: Regional analysis of WSS (Pa) in 4D flow analysis post-vasopressor administration. **S6**: Collagen composition within the sampled tissues via colour deconvolution measurements. **S7**: Elastin composition within the sampled tissues via colour deconvolution measurements. **S8**: Immunohistochemistry results reporting on the percentage of collagen types I in all tissue samples. **S9**: Immunohistochemistry results reporting on the percentage of collagen types III in all tissue samples. **S10**: Immunohistochemistry results reporting on the percentage of collagen types IV in all tissue samples.

## Data Availability

All data incorporated into manuscript.

## Abbreviations

MRI: Magnetic resonance imaging
4D: 4-Dimensional
WSS: Wall sheer stress
PIRL: Preclinical, Imaging and Research Laboratories
CPB: Cardiopulmonary bypass
ROSC: Return of spontaneous circulation
TAA: Thoracic abdominal aneurysm
MMP: Matrix metallopeptidases
TIMP: Tissue inhibitor of metalloproteinases
PGD: Primary graft dysfunction
FiO2: Fraction of inspired oxygen
SvO2: Mixed venous oxygen saturation
PaO2: Partial pressure of oxygen
ROI: Region of interest
SVC: Superior Vena Cava
EVG: Van Gieson’s stain
WIP: Work in progress
