# Supplementary material for: The susceptibility of the aortic root: porcine aortic rupture testing under cardiopulmonary bypass
Source: J Cardiothorac Surg. 2021 Oct 3;16:283. doi: 10.1186/s13019-021-01667-9 (PMC8489069; doi:10.1186/s13019-021-01667-9)
Supplement: Supplementary file 1 — Additional file 1.S1: Summary of radiological results following noradrenaline administration and 4D flow MRI imaging. S2: Regional analysis of Flow (cm/s) in 4D flow analysis pre-vasopressor administration. S3: Regional analysis of Flow (cm/s) in 4D flow analysis post vasopressor administration. S4: Regional analysis of WSS (Pa) in 4D flow analysis pre-vasopressor administration. S5: Regional analysis of WSS (Pa) in 4D flow analysis post-vasopressor administration. S6: Collagen composition within the sampled tissues via colour deconvolution measurements. S7: Elastin composition within the sampled tissues via colour deconvolution measurements. S8: Immunohistochemistry results reporting on the percentage of collagen types I in all tissue samples. S9: Immunohistochemistry results reporting on the percentage of collagen types III in all tissue samples. S10: Immunohistochemistry results reporting on the percentage of collagen types IV in all tissue samples. [file 13019_2021_1667_MOESM1_ESM.docx]

**Supplementary tables**

| **Swine number** | **Region of aorta** | **Analysis number** | **Mean/Max Peak velocity pre vasopressor (cm/s)** | **Mean/Max peak velocity post vasopressor (cm/s)** | **Mean/Max WSS pre vasopressor (Pa)** | **Mean/Max WSS post vasopressor (Pa)** |
| --- | --- | --- | --- | --- | --- | --- |
| **1** | **Root** | **1** | 79.83 | 52.41 | 0.09 | 0.28 |
|  |  | **2** | 51.96 | 53.01 | 0.10 | 0.29 |
|  | **Proximal Ascending Aorta** | **1** | 98.78 | 76.99 | 0.11 | 0.40 |
|  |  | **2** | 54.54 | 83.04 | 0.08 | 0.40 |
|  | **Middle Ascending Aorta** | **1** | 95.27 | 98.78 | 0.14 | 0.48 |
|  |  | **2** | 77.52 | 96.62 | 0.11 | 0.45 |
|  | **Distal Ascending Aorta** | **1** | 52.41 | 95.27 | 0.13 | 0.39 |
|  |  | **2** | 110.26 | 102.21 | 0.13 | 0.42 |
| **2** | **Root** | **1** | 63.42 | 131.70 | 0.11 | 0.38 |
|  |  | **2** | 54.02 | 65.91 | 0.26 | 0.38 |
|  | **Proximal Ascending Aorta** | **1** | 74.53 | 146.69 | 0.21 | 0.49 |
|  |  | **2** | 87.20 | 89.71 | 0.46 | 0.49 |
|  | **Middle Ascending Aorta** | **1** | 99.92 | 184.53 | 0.31 | 0.75 |
|  |  | **2** | 107.32 | 113.69 | 0.62 | 0.75 |
|  | **Distal Ascending Aorta** | **1** | 102.02 | 186.72 | 0.35 | 0.57 |
|  |  | **2** | 104.44 | 101.68 | 0.43 | 0.57 |
| **3** | **Root** | **1** | 52.37 | 62.32 | 0.20 | 0.26 |
|  |  | **2** | 51.04 | 54.73 | 0.20 | 0.30 |
|  | **Proximal Ascending Aorta** | **1** | 73.43 | 94.47 | 0.33 | 0.43 |
|  |  | **2** | 74.93 | 88.95 | 0.31 | 0.45 |
|  | **Middle Ascending Aorta** | **1** | 83.09 | 105.93 | 0.37 | 0.58 |
|  |  | **2** | 88.32 | 98.03 | 0.40 | 0.58 |
|  | **Distal Ascending Aorta** | **1** | 78.34 | 99.95 | 0.24 | 0.32 |
|  |  | **2** | 77.23 | 98.24 | 0.25 | 0.37 |
| **4** | **Root** | **1** | 52.85 | 67.45 | 0.33 | 0.46 |
|  |  | **2** | 53.78 | 59.31 | 0.32 | 0.40 |
|  | **Proximal Ascending Aorta** | **1** | 69.98 | 88.21 | 0.50 | 0.67 |
|  |  | **2** | 70.11 | 66.38 | 0.51 | 0.64 |
|  | **Middle Ascending Aorta** | **1** | 82.58 | 93.25 | 0.62 | 0.82 |
|  |  | **2** | 81.45 | 88.99 | 0.60 | 0.81 |
|  | **Distal Ascending Aorta** | **1** | 95.40 | 102.49 | 0.55 | 0.80 |
|  |  | **2** | 94.40 | 102.78 | 0.55 | 0.82 |
| **5** | **Root** | **1** | 86.29 | 75.83 | 0.28 | 0.31 |
|  |  | **2** | 72.25 | 77.78 | 0.39 | 0.55 |
|  | **Proximal Ascending Aorta** | **1** | 84.10 | 85.03 | 0.44 | 0.60 |
|  |  | **2** | 96.32 | 95.79 | 0.31 | 0.61 |
|  | **Middle Ascending Aorta** | **1** | 86.31 | 109.12 | 0.37 | 0.60 |
|  |  | **2** | 79.73 | 103.87 | 0.27 | 0.55 |
|  | **Distal Ascending Aorta** | **1** | 56.38 | 101.28 | 0.26 | 0.48 |
|  |  | **2** | 50.54 | 102.78 | 0.43 | 0.37 |
|  | **Median** |  | **79.04** | **95.53** | **0.31** | **0.48** |

**S1: Summary of radiological results following noradrenaline administration and 4D flow MRI imaging.**

| Subject number | Root | Proximal | Middle | Distal |
| --- | --- | --- | --- | --- |
| 1 | 79.83 | 98.78 | 95.27 | 52.41 |
| 2 | 51.96 | 54.54 | 77.52 | 110.26 |
| 3 | 63.42 | 74.53 | 99.92 | 102.02 |
| 4 | 54.02 | 87.2 | 107.32 | 104.44 |
| 5 | 52.37 | 73.43 | 83.09 | 78.34 |
| 6 | 51.04 | 74.93 | 88.32 | 77.23 |
| 7 | 52.85 | 69.98 | 82.58 | 95.4 |
| 8 | 53.78 | 70.11 | 81.45 | 94.4 |
| 9 | 86.29 | 84.1 | 86.31 | 56.38 |
| 10 | 72.25 | 96.32 | 79.73 | 50.54 |
| **Median** | **53.90** | **74.73** | **84.70** | **86.37** |

**S2: Regional analysis of Flow (cm/s) in 4D flow analysis pre-vasopressor administration**

| Subject number | Root | Proximal | Middle | Distal |
| --- | --- | --- | --- | --- |
| 1 | 52.41 | 76.99 | 98.78 | 95.27 |
| 2 | 53.01 | 83.04 | 96.62 | 102.21 |
| 3 | 131.7 | 146.69 | 184.53 | 186.72 |
| 4 | 65.91 | 89.71 | 113.69 | 101.68 |
| 5 | 62.32 | 94.47 | 105.93 | 99.95 |
| 6 | 54.73 | 88.95 | 98.03 | 98.24 |
| 7 | 67.45 | 88.21 | 93.25 | 102.49 |
| 8 | 59.31 | 66.38 | 88.99 | 102.78 |
| 9 | 75.83 | 85.03 | 109.12 | 101.28 |
| 10 | 77.78 | 95.79 | 103.87 | 102.78 |
| **Median** | **64.12** | **88.58** | **101.33** | **101.95** |

**S3: Regional analysis of Flow (cm/s) in 4D flow analysis post vasopressor administration**

| Subject number | Root | Proximal | Middle | Distal |
| --- | --- | --- | --- | --- |
| 1 | 0.09 | 0.11 | 0.14 | 0.13 |
| 2 | 0.1 | 0.08 | 0.11 | 0.13 |
| 3 | 0.11 | 0.21 | 0.31 | 0.35 |
| 4 | 0.26 | 0.46 | 0.62 | 0.43 |
| 5 | 0.2 | 0.33 | 0.37 | 0.24 |
| 6 | 0.2 | 0.31 | 0.4 | 0.25 |
| 7 | 0.33 | 0.5 | 0.62 | 0.55 |
| 8 | 0.32 | 0.51 | 0.6 | 0.55 |
| 9 | 0.28 | 0.44 | 0.37 | 0.26 |
| 10 | 0.39 | 0.31 | 0.27 | 0.43 |
| **Median** | **0.23** | **0.32** | **0.37** | **0.31** |

**S4: Regional analysis of WSS (Pa) in 4D flow analysis pre-vasopressor administration**

| Subject number | Root | Proximal | Middle | Distal |
| --- | --- | --- | --- | --- |
| 1 | 0.28 | 0.4 | 0.48 | 0.39 |
| 2 | 0.29 | 0.4 | 0.45 | 0.42 |
| 3 | 0.38 | 0.49 | 0.75 | 0.57 |
| 4 | 0.38 | 0.49 | 0.75 | 0.57 |
| 5 | 0.26 | 0.43 | 0.58 | 0.32 |
| 6 | 0.3 | 0.45 | 0.58 | 0.37 |
| 7 | 0.46 | 0.67 | 0.82 | 0.8 |
| 8 | 0.4 | 0.64 | 0.81 | 0.82 |
| 9 | 0.31 | 0.6 | 0.6 | 0.48 |
| 10 | 0.55 | 0.61 | 0.55 | 0.37 |
| **Median** | **0.35** | **0.49** | **0.59** | **0.45** |

**S5: Regional analysis of WSS (Pa) in 4D flow analysis post-vasopressor administration**

| **Swine number** | **Tissue sampled** | **Distal anterior** | **Distal posterior** | **Distal inner** | **Distal outer** | **Middle anterior** | **Middle posterior** | **Middle inner** | **Middle outer** | **Proximal anterior** | **Proximal posterior** | **Proximal inner** | **Proximal outer** | **Aortic root** |
| --- | --- | --- | --- | --- | --- | --- | --- | --- | --- | --- | --- | --- | --- | --- |
| 1(1) | Ascending aorta and aortic root | 2.49 | 7.20 | 0.17 | 1.25 | 11.61 | 2.42 | 5.47 | 2.28 | 1.82 | 3.99 | 6.17 | 12.44 | 1.83 |
| 1(2) | Ascending aorta and aortic root | 3.21 | 7.23 | 1.13 | 1.28 | 10.56 | 2.67 | 5.98 | 3.56 | 1.98 | 4.85 | 7.24 | 10.34 | 1.98 |
| 2(1) | Ascending aorta and aortic root | 3.08 | 1.50 | 7.65 | 2.18 | 3.78 | 5.82 | 0.84 | 4.36 | 2.33 | 4.76 | 9.24 | 9.65 | 0.87 |
| 2(2) | Ascending aorta and aortic root | 3.77 | 1.55 | 7.22 | 2.56 | 3.88 | 5.99 | 1.23 | 4.44 | 2.57 | 4.65 | 8.88 | 9.02 | 1.06 |
| 3(1) | Ascending aorta and aortic root | 3.33 | 5.55 | 1.27 | 2.01 | 4.06 | 5.03 | 5.25 | 3.81 | 2.34 | 4.32 | 7.32 | 9.84 | 3.77 |
| 3(2) | Ascending aorta and aortic root | 4.56 | 5.06 | 1.85 | 2.32 | 4.32 | 5.55 | 5.91 | 4.00 | 2.21 | 4.74 | 9.35 | 9.13 | 3.02 |
| 4(1) | Ascending aorta and aortic root | 3.75 | 6.00 | 2.75 | 2.67 | 3.72 | 6.78 | 5.12 | 4.76 | 2.56 | 4.54 | 8.55 | 8.34 | 2.94 |
| 4(2) | Ascending aorta and aortic root | 3.75 | 6.11 | 2.69 | 2.11 | 3.91 | 5.10 | 5.87 | 4.12 | 2.75 | 3.87 | 8.56 | 8.75 | 2.64 |
| 5(1) | Ascending aorta and aortic root | 4.01 | 5.86 | 1.67 | 3.64 | 2.54 | 6.10 | 5.54 | 4.87 | 2.33 | 4.67 | 8.41 | 8.88 | 1.06 |
| 5(2) | Ascending aorta and aortic root | 4.44 | 5.32 | 1.85 | 2.86 | 2.50 | 5.09 | 4.91 | 4.67 | 1.97 | 5.34 | 7.46 | 9.03 | 1.87 |
| **Median** |  | **3.75** | **5.71** | **1.85** | **2.25** | **3.90** | **5.33** | **5.36** | **4.24** | **2.33** | **4.66** | **8.48** | **9.08** | **1.93** |

**S6: Collagen composition within the sampled tissues via colour deconvolution measurements**

| **Swine number** | **Tissue sampled** | **Distal anterior** | **Distal posterior** | **Distal inner** | **Distal outer** | **Middle anterior** | **Middle posterior** | **Middle inner** | **Middle outer** | **Proximal anterior** | **Proximal posterior** | **Proximal inner** | **Proximal outer** | **Aortic root** |
| --- | --- | --- | --- | --- | --- | --- | --- | --- | --- | --- | --- | --- | --- | --- |
| 1(1) | Ascending aorta and aortic root | 9.49 | 14.29 | 28.08 | 17.97 | 13.15 | 10.04 | 32.36 | 18.67 | 12.68 | 15.04 | 21.17 | 24.33 | 16.62 |
| 1(2) | Ascending aorta and aortic root | 10.07 | 14.98 | 27.65 | 15.86 | 13.07 | 10.86 | 32.96 | 17.56 | 11.85 | 15.45 | 22.56 | 23.31 | 15.89 |
| 2(1) | Ascending aorta and aortic root | 5.53 | 18.82 | 15.50 | 27.49 | 7.52 | 14.83 | 16.71 | 18.65 | 25.17 | 11.79 | 27.03 | 16.58 | 13.17 |
| 2(2) | Ascending aorta and aortic root | 7.65 | 17.99 | 14.78 | 28.76 | 8.01 | 16.94 | 15.98 | 19.64 | 23.96 | 11.76 | 26.95 | 17.22 | 12.56 |
| 3(1) | Ascending aorta and aortic root | 9.62 | 14.16 | 16.36 | 25.47 | 12.37 | 11.74 | 27.36 | 19.37 | 14.37 | 14.53 | 21.63 | 17.53 | 20.87 |
| 3(2) | Ascending aorta and aortic root | 10.25 | 15.47 | 15.78 | 18.36 | 11.74 | 13.85 | 26.78 | 18.52 | 13.15 | 15.64 | 21.11 | 19.55 | 19.67 |
| 4(1) | Ascending aorta and aortic root | 11.24 | 15.64 | 13.65 | 18.33 | 13.01 | 13.74 | 20.11 | 17.55 | 13.43 | 15.33 | 23.64 | 20.11 | 34.04 |
| 4(2) | Ascending aorta and aortic root | 13.42 | 14.65 | 15.56 | 18.24 | 15.41 | 13.11 | 21.01 | 17.00 | 12.64 | 16.22 | 24.54 | 20.42 | 33.24 |
| 5(1) | Ascending aorta and aortic root | 12.15 | 14.33 | 23.43 | 19.42 | 14.33 | 15.43 | 19.53 | 18.43 | 12.07 | 16.31 | 24.22 | 21.64 | 26.76 |
| 5(2) | Ascending aorta and aortic root | 13.44 | 14.52 | 23.53 | 17.43 | 13.94 | 14.23 | 19.45 | 18.55 | 12.11 | 17.54 | 21.11 | 23.63 | 26.89 |
| **Median** |  | **10.16** | **14.82** | **16.07** | **18.35** | **13.04** | **13.80** | **20.56** | **18.54** | **12.92** | **15.39** | **23.10** | **20.27** | **20.27** |

**S7: Elastin composition within the sampled tissues via colour deconvolution measurements**

| **Swine number** | **Tissue sampled** | **Distal anterior** | **Distal posterior** | **Distal inner** | **Distal outer** | **Middle anterior** | **Middle posterior** | **Middle inner** | **Middle outer** | **Proximal anterior** | **Proximal posterior** | **Proximal inner** | **Proximal outer** | **Aortic root** |
| --- | --- | --- | --- | --- | --- | --- | --- | --- | --- | --- | --- | --- | --- | --- |
| 1(1) | Ascending aorta and aortic root | 16.04 | 12.12 | 27.87 | 23.20 | 19.53 | 19.73 | 24.14 | 8.31 | 12.01 | 11.64 | 26.54 | 25.64 | 22.93 |
| 1(2) | Ascending aorta and aortic root | 15.00 | 13.11 | 26.87 | 22.87 | 20.64 | 20.63 | 24.07 | 10.63 | 12.11 | 13.21 | 25.22 | 25.86 | 23.52 |
| 2(1) | Ascending aorta and aortic root | 14.84 | 10.28 | 42.56 | 30.60 | 16.84 | 16.60 | 26.33 | 27.99 | 13.70 | 14.49 | 20.61 | 23.58 | 25.20 |
| 2(2) | Ascending aorta and aortic root | 14.88 | 11.95 | 41.52 | 31.82 | 15.74 | 18.76 | 26.04 | 26.78 | 13.06 | 14.92 | 21.53 | 22.67 | 24.53 |
| 3(1) | Ascending aorta and aortic root | 17.32 | 13.63 | 33.33 | 22.53 | 19.80 | 20.03 | 26.02 | 25.93 | 12.03 | 12.36 | 22.28 | 22.82 | 23.52 |
| 3(2) | Ascending aorta and aortic root | 17.63 | 14.65 | 34.11 | 24.54 | 19.33 | 21.49 | 26.26 | 25.29 | 12.83 | 12.20 | 26.27 | 21.68 | 23.32 |
| 4(1) | Ascending aorta and aortic root | 16.31 | 12.66 | 23.01 | 19.72 | 15.01 | 20.38 | 25.28 | 28.02 | 11.46 | 13.27 | 27.27 | 27.57 | 25.83 |
| 4(2) | Ascending aorta and aortic root | 16.33 | 13.83 | 22.02 | 19.55 | 16.22 | 20.10 | 27.02 | 28.93 | 11.44 | 15.47 | 28.10 | 29.37 | 24.53 |
| 5(1) | Ascending aorta and aortic root | 15.00 | 12.52 | 27.92 | 19.21 | 19.03 | 16.81 | 27.25 | 18.30 | 13.10 | 12.17 | 16.81 | 27.94 | 26.42 |
| 5(2) | Ascending aorta and aortic root | 15.92 | 12.44 | 29.92 | 19.42 | 18.11 | 19.11 | 27.22 | 18.92 | 12.26 | 12.71 | 18.81 | 27.24 | 26.53 |
| **Median** |  | **15.98** | **12.59** | **28.92** | **22.70** | **18.57** | **19.88** | **26.15** | **25.61** | **12.19** | **12.96** | **23.75** | **25.75** | **24.53** |

**S8: Immunohistochemistry results reporting on the percentage of collagen types I in all tissue samples.**

| **Swine number** | **Tissue sampled** | **Distal anterior** | **Distal posterior** | **Distal inner** | **Distal outer** | **Middle anterior** | **Middle posterior** | **Middle inner** | **Middle outer** | **Proximal anterior** | **Proximal posterior** | **Proximal inner** | **Proximal outer** | **Aortic root** |
| --- | --- | --- | --- | --- | --- | --- | --- | --- | --- | --- | --- | --- | --- | --- |
| 1(1) | Ascending aorta and aortic root | 15.13 | 10.15 | 21.31 | 16.93 | 11.73 | 7.50 | 35.25 | 21.62 | 10.60 | 6.79 | 16.80 | 15..59 | 23.35 |
| 1(2) | Ascending aorta and aortic root | 16.93 | 11.35 | 20.91 | 16.29 | 11.10 | 6.35 | 33.26 | 21.84 | 10.75 | 7.00 | 16.02 | 14.54 | 22.83 |
| 2(1) | Ascending aorta and aortic root | 16.39 | 10.84 | 18.47 | 16.02 | 10.35 | 10.35 | 16.24 | 20.54 | 11.45 | 8.30 | 15.50 | 16.53 | 20.40 |
| 2(2) | Ascending aorta and aortic root | 16.29 | 13.29 | 19.51 | 15.37 | 11.47 | 10.47 | 17.13 | 19.36 | 10.30 | 9.80 | 16.35 | 15.20 | 24.01 |
| 3(1) | Ascending aorta and aortic root | 17.53 | 11.34 | 18.82 | 18.83 | 12.42 | 10.54 | 25.24 | 23.47 | 9.46 | 7.60 | 15.30 | 11.54 | 26.01 |
| 3(2) | Ascending aorta and aortic root | 17.49 | 10.39 | 18.94 | 18.01 | 12.34 | 10.20 | 25.35 | 22.54 | 9.32 | 7.24 | 15.01 | 11.04 | 25.22 |
| 4(1) | Ascending aorta and aortic root | 20.74 | 10.43 | 19.32 | 17.81 | 17.13 | 13.86 | 25.34 | 17.42 | 11.46 | 6.76 | 18.54 | 12.00 | 24.22 |
| 4(2) | Ascending aorta and aortic root | 19.83 | 11.34 | 23.28 | 18.72 | 16.53 | 11.74 | 26.86 | 15.57 | 12.30 | 6.06 | 17.22 | 12.10 | 19.34 |
| 5(1) | Ascending aorta and aortic root | 17.81 | 12.93 | 23.10 | 16.73 | 15.42 | 12.03 | 23.46 | 23.54 | 10.30 | 6.90 | 13.30 | 15.64 | 28.02 |
| 5(2) | Ascending aorta and aortic root | 15.91 | 11.49 | 18.92 | 15.38 | 15.90 | 10.82 | 24.98 | 23.67 | 10.55 | 6.03 | 13.20 | 15.39 | 22.05 |
| **Median** |  | **17.21** | **11.34** | **19.42** | **16.83** | **12.38** | **10.51** | **25.29** | **21.73** | **10.58** | **6.95** | **15.76** | **14.54** | **23.68** |

**S9: Immunohistochemistry results reporting on the percentage of collagen types III in all tissue samples.**

| **Swine number** | **Tissue sampled** | **Distal anterior** | **Distal posterior** | **Distal inner** | **Distal outer** | **Middle anterior** | **Middle posterior** | **Middle inner** | **Middle outer** | **Proximal anterior** | **Proximal posterior** | **Proximal inner** | **Proximal outer** | **Aortic root** |
| --- | --- | --- | --- | --- | --- | --- | --- | --- | --- | --- | --- | --- | --- | --- |
| 1(1) | Ascending aorta and aortic root | 16.39 | 9.17 | 13.21 | 13.29 | 12.00 | 28.70 | 22.49 | 26.00 | 22.21 | 19.32 | 16.34 | 19.50 | 15.97 |
| 1(2) | Ascending aorta and aortic root | 16.03 | 10.24 | 13.21 | 13.35 | 11.89 | 27.39 | 21.54 | 25.00 | 23.42 | 18.30 | 16.23 | 18.03 | 15.13 |
| 2(1) | Ascending aorta and aortic root | 15.46 | 11.64 | 14.20 | 14.42 | 13.52 | 24.75 | 20.30 | 24.56 | 17.34 | 23.34 | 13.01 | 19.34 | 16.42 |
| 2(2) | Ascending aorta and aortic root | 15.04 | 11.72 | 15.20 | 14.13 | 13.20 | 23.03 | 24.43 | 23.50 | 18.32 | 22.54 | 17.39 | 18.43 | 15.32 |
| 3(1) | Ascending aorta and aortic root | 16.42 | 10.45 | 13.02 | 17.13 | 11.56 | 22.53 | 22.43 | 24.46 | 25.23 | 22.83 | 16.01 | 15.89 | 18.32 |
| 3(2) | Ascending aorta and aortic root | 16.12 | 10.20 | 14.20 | 16.5 | 11.39 | 24.03 | 23.11 | 24.56 | 22.49 | 21.02 | 16.40 | 16.74 | 14.24 |
| 4(1) | Ascending aorta and aortic root | 18.50 | 13.23 | 13.02 | 11.23 | 12.03 | 26.04 | 22.03 | 24.56 | 21.23 | 19.34 | 15.03 | 19.34 | 15.63 |
| 4(2) | Ascending aorta and aortic root | 19.42 | 13.10 | 14.93 | 11.00 | 12.20 | 25.94 | 19.44 | 24.09 | 28.34 | 34.39 | 15.30 | 19.00 | 14.24 |
| 5(1) | Ascending aorta and aortic root | 14.24 | 11.50 | 14.20 | 15.20 | 12.56 | 25.38 | 20.79 | 24.40 | 22.54 | 33.32 | 14.03 | 18.42 | 16.40 |
| 5(2) | Ascending aorta and aortic root | 14.10 | 12.39 | 13.20 | 15.60 | 12.57 | 25.11 | 22.20 | 22.03 | 22.12 | 21.20 | 16.03 | 21.32 | 17.22 |
| **Median** |  | **16.10** | **11.57** | **14.17** | **14.28** | **12.12** | **25.26** | **22.12** | **24.51** | **22.35** | **17.85** | **16.02** | **18.72** | **15.80** |

**S10: Immunohistochemistry results reporting on the percentage of collagen types IV in all tissue samples.**
